# Greater Efficacy of Black Ginseng (CJ EnerG) over Red Ginseng against Lethal Influenza A Virus Infection

**DOI:** 10.3390/nu11081879

**Published:** 2019-08-13

**Authors:** Eun-Ha Kim, Son-Woo Kim, Su-Jin Park, Semi Kim, Kwang-Min Yu, Seong Gyu Kim, Seung Hun Lee, Yong-Ki Seo, Nam-Hoon Cho, Kimoon Kang, Do Y. Soung, Young-Ki Choi

**Affiliations:** 1 College of Medicine and Medical Research Institute, Chungbuk National University, Cheongju 28644, Korea; 2 The Institutes of Food, CJ CheilJedang, Suwon 16495, Korea; 3 ID Bio Corporation, Cheongju 28370, Korea

**Keywords:** black ginseng, oral administration, influenza A virus, cytokines, antiviral

## Abstract

Black ginseng (BG, CJ EnerG), prepared via nine repeated cycles of steaming and drying of fresh ginseng, contains more accessible acid polysaccharides and smaller and less polar ginsenosides than red ginseng (RG) processed only once. Because RG exhibits the ability to increase host protection against viral respiratory infections, we investigated the antiviral effects of BG. Mice were orally administered either BG or RG extract at 10 mg/kg bw daily for two weeks. Mice were then infected with a A(H1N1) pdm09 (A/California/04/2009) virus and fed extracts for an additional week. Untreated, infected mice were assigned to either the negative control, without treatments, or the positive control, treated with Tamiflu. Infected mice were monitored for 14 days to determine the survival rate. Lung tissues were evaluated for virus titer and by histological analyses. Cytokine levels were measured in bronchoalveolar lavage fluid. Mice treated with BG displayed a 100% survival rate against infection, while mice treated with RG had a 50% survival rate. Further, mice treated with BG had fewer accumulated inflammatory cells in bronchioles following viral infection than did mice treated with RG. BG also enhanced the levels of GM-CSF and IL-10 during the early and late stages of infection, respectively, compared to RG. Thus, BG may be useful as an alternative antiviral adjuvant to modulate immune responses to influenza A virus.

## 1. Introduction

Influenza viruses are RNA viruses of seven different genera in the family Orthomyxoviridae: Influenza viruses A, B, C, and D, Quaranjavirus, Thogotovirus, and Isavirus [1,2]. Of these, the influenza type A virus is responsible for the most common outbreaks of clinical respiratory diseases. These include all the human influenza pandemics such as the 1918 Spanish flu, 1957 Asian flu, 1968 Hong Kong flu, and most recently, the 2009 swine flu [3,4,5]. The Center for Disease Control and Prevention (CDC) in the United States recently announced influenza surveillance reports based on data collected from October 2018 through May 2019. In this time frame, influenza caused an estimated 37.4 to 42.9 million flu illnesses and between 36,400 and 61,200 deaths [6].

Influenza vaccination and antiviral treatments have been used as the most effective methods to prevent the spread and reduce the mortality of novel and potentially pandemic influenza viruses. Inactivated trivalent and quadrivalent influenza vaccines and a live attenuated influenza vaccine are commonly used [7]. Licensed influenza antiviral drugs include an influenza A virus M2 ion channel blocker (Amantadine, Symmetrel^®^, Endo Pharmaceuticals), and influenza A and B virus neuraminidase inhibitors (Oseltamivir Tamiflu, Roche Laboratories Inc; Zanamivir, Relenza, GlaxoSmithKline) [8,9,10,11]. However, due to the increased frequency of viral resistance to vaccines and drugs caused by the rapid mutation of influenza virus genomes, alternative anti-influenza therapeutic strategies are needed [12].

For this reason, many researchers have investigated potent antiviral activities of natural compounds or extracts [13,14,15,16]. We previously investigated the anti-influenza effects of KIOM-C, which is an herbal compound mixture of Scutellariae Radix, Glycyrrhizae Radix, Paeoniae Radix Alba, Angelicae Gigantis Radix, Platycodon Grandiflorum, Zingiber Officinale, Lonicera Japonica Thunberg, and Saposhnikovia Divaricata Schiskin. We demonstrated that KIOM-C decreases the viral burden in the respiratory tracts of both mice and ferrets infected with influenza A virus [17]. In particular, red ginseng (RG) has been reported to prevent lung immunopathology, leading to increased survival rates against various subtype A influenza virus (H1N1, H5N1, and H3N2) infections in mice [18,19,20,21,22,23]. These studies were supported by a randomized and double-blind clinical trial with healthy subjects demonstrating that the frequency of acute respiratory illness in the RG group was significantly lower than in the placebo group [24].

**Figure 1:**
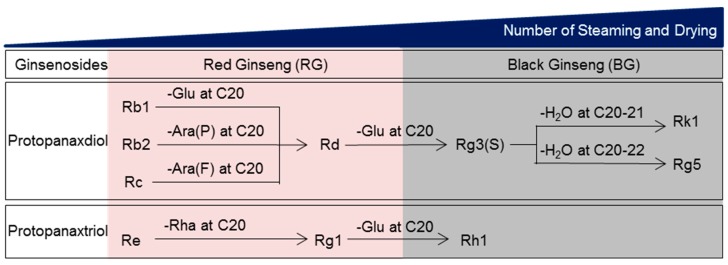
Transformation of the ginsenoside profile of ginseng with increased numbers of steaming and drying cycles. There are two types of ginsenosides: protopanaxadiol-type saponins (e.g., Rb1, Rb2, Rc, Rd, Rg3, Rg5, and Rk1) with sugar moieties attached to hydroxyl groups at C3 and C20 and protopanaxatriol type saponins (e.g., Re, Rg1, and Rh1) with sugar moieties attached to hydroxyl groups at C3, C6, and C20. The outer residues from position C20 of Rb1, Rb2 and Rc are glucose, arabinose (pyranose form), and arabinose (furanose form), respectively. These outer residues are removed to achieve Rd. The remaining glucose of Rd at C20 can be deleted to form Rg3. Sequentially, Rk1 with the double bond at C20-21 and Rg5 with a double bond at C20-22 are derived from Rg3 by dehydration at C20. Re, a protopanaxatriol type, can also be transformed to Rg1 after deletion of the rhamnose residue at C6. The outer glucose residue of Rg1 at C20 is removed to form Rh1. Glu: glucose; Ara(P): arabinose (pyranose form); Ara(F): arabinose (furanose form); Rha: rhamnose.

Unlike RG that is prepared via one-time steaming and drying of fresh ginseng (Panax ginseng C.A. Meyer), black ginseng (BG) is made by repeating the same process nine times. During this process, ginsenosides, the pharmacological components found in ginseng, are transformed into smaller and less polar molecules by removing sugars and dehydrating at C-3, C-6, or C-20 (Figure 1) [25]. Steaming also leads to a significant increase in acid polysaccharides and phenolic compounds [25,26]. Further, because BG has substantially different components compared to RG, we established that BG is a safe functional ingredient and registered it as a new dietary ingredient with the Food and Drug Administration in the United States in 2016 (CJ EnerG: Notification Number, 897). However, the protective role of this ingredient against viral infection has not been investigated. Therefore, we evaluated the antiviral properties of BG (CJ EnerG) and compared them with those of RG.

## 2. Materials and Methods

### 2.1. Preparation of BG (CJ EnerG) Extract

BG and RG extracts were provided by CJ CheilJedang Corporation (Suwon, Korea). Briefly, BG and RG were subjected to extraction by adding a solution of ethanol and water in a heat reflux extraction system. To generate the final BG and RG products for use in the animal studies, extracts were filtered and concentrated to 70 Brix by removing ethanol and water.

### 2.2. Analysis of Acid Polysaccharides

Pulverized BG and RG powders (400 mg each) were extracted with 10 mL of distilled water at 90 °C for 3 h. Subsequently, the extraction was centrifuged at 3000 rpm for 10 min followed by the addition of 1 mL of the supernatant to 4 mL of ethanol and centrifugation at 3000 rpm for 10 min. After the supernatant was removed, the precipitant was dissolved in a mixture of n-butanol–chloroform and water (1:4, v/v) and then centrifuged at 3000 rpm for 10 min. The final sample was made by adding 4 mL of distilled water to this precipitate. Then a mixture of 50 μL of the sample, 50 μL of distilled water, 50 μL of 0.1% carbazole–ethanol reagent, and 600 μL of sulfuric acid was placed in a 96 well-plate and analyzed at 530 nm using a multireader (Thermo scientific VARIOSKAN LUX, Vantaa, Finland). The amount of acid polysaccharides was calculated based on the calibration curve generated using galacturonic acid as the standard [27].

### 2.3. Measurements of Ginsenosides

Based on a modified method of Jin et al. [25], 2.5 g of each extract was dissolved in 50 mL of 70% methanol at room temperature for 30 min using an ultrasonic generator (Branson 8510, Danbury, CT, USA). The solution was centrifuged at 1600 g for 10 min (Labogene 1248R, Lynge, Denmark) at 4 °C. The supernatant was then filtered using a 0.45 μm membrane filter (Pall Corporation, Port Washington, NY 11050, USA) and was resolved on a C18 column (Venusil XBP C18, 4.5 × 250 mm, ID 5 μm, 100 Å) with acetonitrile and distilled water. The amount of each ginsenoside was then measured using an HPLC with DAD (Agilent 1260, Palo Alto, CA, USA) analysis.

### 2.4. Virus

An influenza A strain, A/California/04/2009 (CA04, H1N1) isolated in 2009, was propagated for 48 h at 37 °C in the allantoic cavities of specific-pathogen-free 10-day-old chicken eggs. Clarified allantoic fluids were aliquoted and then stored at −70 °C until use. The virus titer was calculated as 50% of the tissue culture infectious dose (TCID_50_) in Madin-Darby Canine Kidney (MDCK) cells by the method of Reed and Muench [28]. MDCK cells obtained from the American Type Culture Collection (ATCC) were maintained in Eagle’s minimal essential medium (EMEM) (LONZA, Inc., Allendale, NJ, USA) supplemented with 5% fetal bovine serum (LONZA, Inc., Allendale, NJ, USA) and 1% penicillin/streptomycin (Gibco-Invitrogen, Inc., Carlsbad, CA, USA).

### 2.5. Mice and Treatments

**Figure 2:**
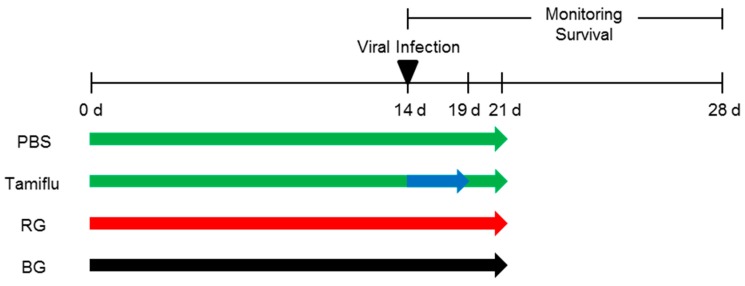
Schematic diagram of animal experiments. Mice were orally administered either red ginseng (RG) or black ginseng (BG) (10 mg/kg bw daily) for 14 days. After challenge with A/California/04/2009 virus, mice continuously received either RG or BG for an additional week. As a negative control, mice that received phosphate buffered saline (PBS) daily for 14 days were also infected with virus. The positive control group was treated with Tamiflu daily for 5 days post-infection and then with PBS for 2 additional days. All mice were monitored for 14 days post-infection to measure survival.

Five-week-old BALB/c female mice were purchased from Samtaco (Pyungteack, Korea). After one-week of acclimation, mice were orally administered 10 mg/kg of body weight of either BG or RG extract in a total volume 200 µl for two weeks (Figure 2). Doses of BG and RG for treatments were determined based on a previous report [29] and confirmed by our preliminary study. Mice were then intranasally inoculated with five times the 50% mouse lethal dose (MLD_50_) of A/California/04/2009 (105.5 TCID_50_/_mL_) in a volume of 30 µl and treated with the extracts for an additional week. Untreated, infected mice were assigned either a negative control treated with phosphate buffered saline (PBS) or a positive control treated with Tamiflu (2 mg/kg bw daily) for 5 days. Uninfected mice were also included as an intact control group. Mouse studies were conducted in strict accordance and adherence to relevant policies regarding animal handling as mandated under the Guidelines for Animal Use and Care of the Korea Center for Disease Control (K-CDC). The study was approved by the Medical Research Institute (approval number CBNUA-1196-18-01).

### 2.6. Measurement of Survival

Following infection, mice were monitored for 14 days to determine the survival rate. Mice showing more than 25% loss of body weight were considered to be dying and were euthanized.

### 2.7. Determination of Lung Viral Titers

Lung tissues (*n* = 6) from each group were aseptically collected at 1, 3, 5, and 7 days post-infection (dpi), and homogenized in EMEM containing antibiotics (0.1% penicillin-streptomycin; Gibco-Invitrogen, Inc., Carlsbad, CA, USA). Uninfected lung samples were also used as an intact control. Ten-fold serial dilutions of supernatants were added in quadruplicate to a monolayer of MDCK cells seeded in 96-well cell culture plates. The cells were allowed to absorb virus in the supernatants of the homogenized samples for 1 h at 37 °C in a 5% CO_2_ incubator. After supernatants were removed, the cells were incubated with fresh EMEM and 1 μg/mL N-tosyl-l-phenylalanine chloromethyl ketone -trypsin for 48 h at 37 °C in a 5% CO_2_ incubator. The cytopathic effect of the virus was observed daily, and the viral titer was determined by the hemagglutination test using 0.5% turkey red blood cells.

### 2.8. Histopathological Assays

The lungs of mice infected with A/California/04/2009 virus were harvested at 5 dpi. The samples were fixed in 10% neutral-buffered formalin and embedded in paraffin. Deparaffinized histological sections stained with hematoxylin and eosin (H&E) were viewed and captured using an Olympus IX 71 (Olympus, Tokyo, Japan) microscope.

### 2.9. Measurements of Cytokines

Bronchoalveolar lavage fluid (BALF) samples were isolated from mouse lungs at 1, 3, 5, and 7 dpi. BALF from uninfected mice was used as an intact control. Collected samples were centrifuged at 12,000 rpm for 5 min at 4 °C, aliquoted, and stored at −70 °C until the analysis. BALF samples (20 μL) were incubated with antibody-coupled beads specific for Interleukin 1 beta (IL-1β), IL-2, IL-10, tumor necrosis factor-alpha (TNF-α), interferon-gamma (IFN-γ), and granulocyte-macrophage colony-stimulating factor (GM-CSF). The complexes were washed, incubated with biotinylated detection antibody and streptavidin–phycoerythrin. Cytokine levels in BALF samples were then determined using a multiplex array reader from Luminex™ Instrumentation System (Bio-Plex Workstation, Bio-Rad Laboratories, Hercules, CA, USA).

### 2.10. IgG Assay

ELISA plates (Immunlon 4 HBX, Thermo Scientific, Waltham, MA, USA) were coated with purified virus (1 mg/mL) diluted in carbonate/bicarbonate coating buffer (pH 9.4; Sigma-Aldrich, St. Louis, MO, USA) overnight at 4 °C. The plates were then blocked with PBS containing 0.1% Tween 20 (PBST) and 5% nonfat dry milk powder for 1 h at room temperature (RT). Serial dilutions (1:10) of the serum from each mouse in PBST containing 2% nonfat dry-milk powder were added to plates and incubated for 1 h at RT. After extensive washing with PBST (3 times, 100 μL/well), the plates were incubated for 1 h at RT with an antimouse horseradish peroxidase-conjugated immunoglobulin G (IgG, Abcam, Cambridge, MA, USA) diluted in PBST containing 2% nonfat dry-milk powder. After three more washes with PBST, the plates were overlaid with o-phenylenediamine dihydrochloride (SigmaFast OPD; Sigma-Aldrich, St. Louis, MO, USA) substrate. Reactions were stopped using 3 M HCl. The concentrations of IgG in serum were measured at an optical density of 450 nm on an iMark Microplate Absorbance Reader (iMark Microplate Absorbance Reader, Bio-Rad Laboratories, Hercules, CA, USA).

### 2.11. Hemagglutination Inhibition (HI) Assay and Plaque Reduction Assay

To assess the antiviral effects of BG, a hemagglutination inhibition assay was performed. Briefly, in 96-well plates, RG and BG (10 mg/mL) were serially diluted in PBS at two-fold increments in 25 μL volume, and equal volumes of influenza A virus (4-8 HAU in 50 μL) were added to each well. The plate was incubated at room temperature for 60 min. Hemagglutination inhibition results appeared as dots in the centers of the wells.

For plaque reduction assays, confluent monolayers of MDCK cells were seeded in 6-well tissue culture plates (1 × 106 cells/well) and incubated at 37 °C under 5% CO_2_. RG or BG was diluted at various concentrations (0.1, 0.25, and 0.5 mg/mL) into the media for pretreatment (2 h before virus infection) or the cells were infected with 100 pfu of A/California/04/2009 for 1 h, the inoculum was removed, and test media containing RG, BG, or Oseltamivir (10 uM) were added post-treatment. After 1 h incubation, the supernatant was removed and replaced with overlay medium (EMEM containing 1 ug/mL N-tosyl-l-phenylalanine chloromethyl ketone (TPCK)–trypsin, 0.7% agarose, without serum). Cells were then incubated for 48 h at 37 °C in 5% CO_2_. Test plates were then fixed in a 10% formaldehyde solution for 30 min. Agarose was removed, and cells were stained with a 1% (w/v) crystal violet solution. The plaques were counted by visual examination, and the percentage of plaque reduction was calculated as relative to the control without RG or BG.

### 2.12. Statistical Analysis

To assess the effects of treatment and time, a two-way analysis of variance (ANOVA) test was run. When ANOVA indicated any significant difference among the means (*p* < 0.05), Fisher’s least significant difference test, without correction for multiple comparison, was used to determine which means were significantly different (*p* < 0.05). * Indicates *p* < 0.05 vs. PBS, # indicates *p* < 0.05 vs. Tamiflu, and $ indicates *p* < 0.05 vs. RG. All statistical analyses were performed using GraphPad Prism version 8.00 for Windows (GraphPad Software, La Jolla, CA, USA).

## 3. Results

### 3.1. The Components of BG Differ from Those of RG

To identify differences in the composition of RG and BG, we measured the amounts of acid polysaccharides and major ginsenosides (Table 1). BG contained at least 7-fold higher acid polysaccharides than RG (2.37 vs. 0.37 mg/g extract). RG contained 4.91 mg/g of Rb1, 2.21 mg/g of Rb2, 3.23 mg/g of Rc, 1.75 mg/g of Rd, 3.74 mg/g of Re, 1.29 mg/g of Rg1, 0.42 mg/g of Rg3 (S), 0.12 mg/g of Rk1, 0.46 mg/g of Rg5, and 0.43 mg/g of Rh1. BG contained 0.83 mg/g of Rb1, 1.34 mg/g of Re, 4.12 mg/g of Rg3 (S), 4.75 mg/g of Rk1, 4.54mg/g of Rg5, and 0.94 mg/g of Rh1. Further, Rb2, Rc, Rd, and Rg1 were not detected in BG. These data indicate that BG considerably differs from RG in the amount of acid polysaccharides and the profile of ginsenosides.

### 3.2. BG Protects Mice Against Lethal Influenza A Virus Infection

**Figure 3:**
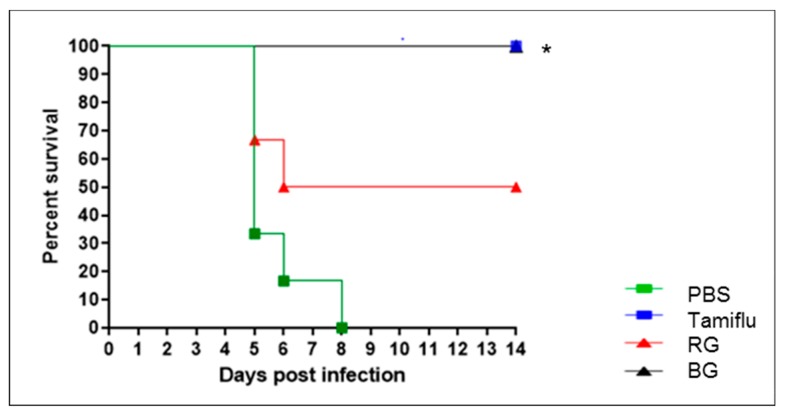
Treatment with BG results in a higher survival rate in influenza virus-infected mice than does treatment with RG. Survival was monitored for 14 days after influenza A virus infection in PBS-, Tamiflu-, RG-, and BG-treated mice (*n* = 6 per group). * *p* < 0.05 vs. PBS.

To examine the antiviral effects of BG, mice (n = 6 per group) were orally administered 10 mg/kg bw of either RG or BG daily for a total of three weeks. Following two weeks of treatment, the mice were infected with a lethal dose of A/California/04/2009 virus and monitored for survival for two weeks. Additional infected mice were assigned to either the negative control, treated with PBS, or the positive control, treated with Tamiflu (Figure 3).

None of the mice treated with Tamiflu succumbed to infection (*p* < 0.05 vs. PBS). On the other hand, the PBS-treated negative control group showed more than 25% weight loss (data not shown), and all mice were dead by 8 dpi. Mice treated with RG showed a 50% survival rate against viral infection, while BG protected all infected mice from virus-associated death, which was a significant increase compared to the PBS-treated group (*p* < 0.05). This result demonstrates that administration of BG provides increased protection of the host against influenza virus compared to treatment with RG.

### 3.3. Administration of BG Results in Reduced Viral Burden and Lung Histopathology Following Lethal Influenza A Virus Infection

**Figure 4:**
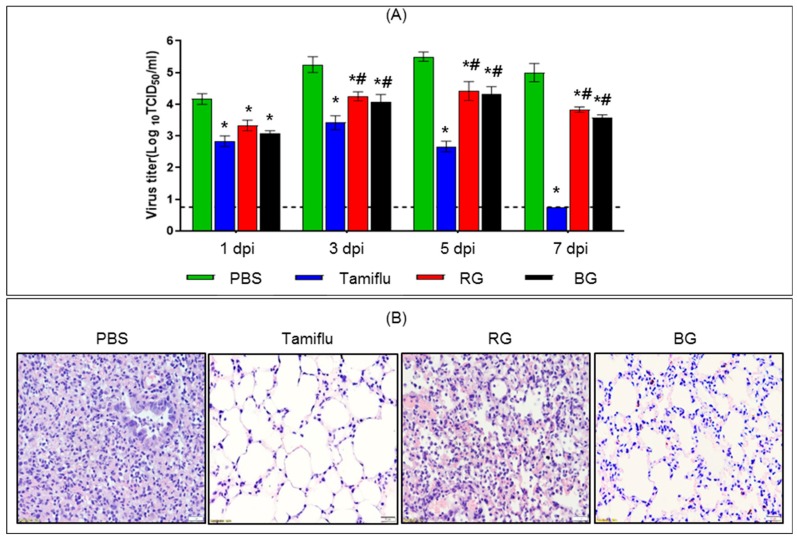
Treatment with BG improves antiviral activity and prevents histopathological alterations following viral infection. (**A**) PBS-, Tamiflu-, RG-, and BG- treated mice were euthanized to collect lung tissues at 1, 3, 5, and 7 dpi. Uninfected mouse lungs were also isolated to use as an intact control (dotted line). Lung viral titer was determined in homogenized tissues by the hemagglutination test. Values are mean (n = 6 per group at each time point) ± SEM. *, *p* < 0.05 vs. PBS; #, *p* < 0.05 vs. Tamiflu. (**B**) At 5 dpi, paraffin-embedded lung samples were prepared from infected mice treated with either PBS, Tamiflu, RG, or BG. Representative histological sections of lung tissues stained with H&E to visualize inflammatory lesions (magnification: × 100).

To assess the inhibitory effects of BG on viral growth, we measured the virus titer in infected mouse lungs (n = 3 per group) at 1, 3, 5, and 7 dpi (Figure 4A). Samples from uninfected mice were used as an intact control. Viral titers in PBS-treated mice were 4.1, 5.3, 5.5, and 5.0 log10 TCID_50_/_mL_ at 1, 3, 5, and 7 dpi, respectively. On the other hand, the viral load in Tamiflu-treated mice was lower than in the PBS-treated group at all time points (*p* < 0.05). Moreover, the Tamiflu-treated group showed no viral burden at 7 dpi. The RG-treated group showed 6.3, 10.0, 12.3, and 14.7 times lower virus load at 1, 3, 5, and 7 dpi, respectively, than the PBS-treated group (*p* < 0.05). Similarly, the BG-treated group displayed 12.5, 17.7, 15.8, and 26.3 times lower virus load at 1, 3, 5, and 7 dpi, respectively, than the PBS-treated group (*p* < 0.05). However, there was no significant difference in the degree of decrease of the viral titer between RG- and BG-treated groups.

To confirm whether the reduced virus titer in BG-treated groups was associated with decreased virus-mediated lung pathology, we examined lungs from each group at 5 dpi (Figure 4B). Typically, influenza A virus replication is accompanied by infiltration of immune cells into lung tissues of the infected host [30,31]. PBS-treated mice showed induction of widespread inflammatory processes in the lung. By contrast, minimal histological alterations were observed in the lung tissue of Tamiflu-treated mice. RG-treated mice showed modest alleviation of infection-induced inflammation and disruption of the membrane barrier of the lung alveolar septum. Interestingly, BG-treated mice showed considerably reduced lung inflammation and pneumonia compared with that of PBS- and RG-treated groups. Our results suggest that oral administration of BG improves antiviral activity and prevents histopathological alterations against lethal influenza A virus.

### 3.4. BG Induces Cytokine Production in Infected Mice

**Figure 5:**
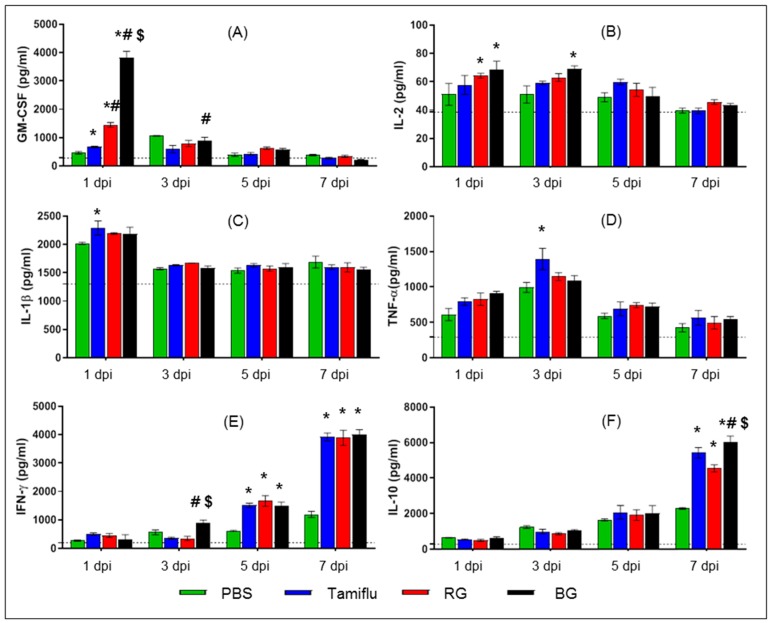
BG treatment further enhances production of cytokines in BALF. At 1, 3, 5, and 7 dpi BALF samples were harvested from PBS-, Tamiflu-, RG-, and BG- treated mouse lungs. Bronchoalveolar lavage fluid (BALF) samples were also isolated from uninfected mice for use as an intact control (dotted line). Cytokine production was analyzed in lung BALF by BioPlex analysis. (**A**) Granulocyte-macrophage colony-stimulating factor (GM-CSF), (**B**) Interleukin 2 (IL-2), (**C**) IL-1β, (**D**) tumor necrosis factor-alpha (TNF-α), (**E**) interferon-gamma (IFN-γ), and (**F**) IL-10. Values are the mean (n = 6 per group at each time point) ± SEM. *, *p* < 0.05 vs. PBS; #, *p* < 0.05 vs. Tamiflu; $, *p* < 0.05 vs. RG.

Cytokines including GM-CSF, IL-2, IL-1β, TNF-α, IFN-γ, and IL-10 are key molecules that regulate innate and adaptive immune responses to viral infection [32,33]. To determine whether BG affected the production of these cytokines during influenza A virus infection, we measured them in local BALF at 1, 3, 5, and 7 dpi (Figure 5). BALF samples collected from lungs of uninfected mice were also used as a control.

At the early stage of infection (1 dpi), Tamiflu-, RG-, and BG-treated mice displayed higher levels of GM-CSF than PBS-treated mice (*p* < 0.05) (Figure 5A). In particular, BG-treated mice showed the highest levels of GM-CSF, elevated 2.6, 5.7. and 8.3-fold over RG-, Tamiflu-, and PBS-treated mice, respectively (*p* < 0.05). BG also induced higher levels of GM-CSF than Tamiflu at 3 dpi (*p* < 0.05). By contrast, no differences in the levels of GM-CSF were observed among the groups at the late stage of infection (5 and 7 dpi).

The levels of IL-2 were also increased in RG- and BG-treated mice at 1 dpi and in BG-treated mice at 3 dpi compared to PBS-treated mice (*p* < 0.05) (Figure 5B). However, no significant difference was observed among groups at 5 or 7 dpi.

Increased levels of IL-1β and TNF-α were observed in Tamiflu-treated mice compared to PBS-treated mice at 1 and 3 dpi, respectively (*p* < 0.05) (Figure 5C,D). Aside from these differences, the levels of IL-1β and TNF-α were consistent among the groups at all other time points.

While the levels of IFN-γ were comparable among the groups at 1 dpi, there was an increase in this cytokine in BG-treated mice compared to both Tamiflu- and RG-treated mice at 3 dpi (*p* < 0.05) (Figure 5E). However, this dramatic induction of IFN-γ was comparable among Tamiflu-, RG-, and BG-treated mice at both 5 and 7 dpi.

There were no differences in levels of IL-10 in any of the groups at 1, 3, or 5 dpi (Figure 5F), Tamiflu-, RG-, and BG-treated mice displayed higher levels of IL-10 than PBS-treated mice (*p* < 0.05) at 7 dpi. However, mice treated with BG showed the highest level of IL-10 with a significant increase over mice treated with either Tamiflu or RG (*p* < 0.05).

These results suggest that BG can modulate the secretion of cytokines during the immune response to influenza A virus in mice.

### 3.5. BG Does Not Affect the Development of Normal Influenza-Specific Antibodies

**Figure 6:**
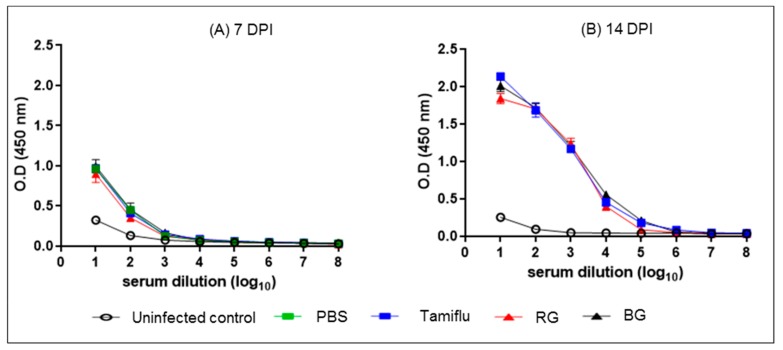
BG treatment does not disturb the normal development of IgG following the first virus inoculation. PBS-, Tamiflu-, RG-, and BG- treated mice were euthanized at 7 and 14 dpi to collect sera. Serum was also isolated from uninfected mice for use as an intact control. Anti-influenza A virus IgG titers were measured in sera by ELISA. Data are representative of three independent experiments. Values are the mean (n = 6 per group at each time point) ± SEM.

To examine the effect of BG on influenza virus-induced adaptive immunity, we measured influenza virus-specific IgG in sera collected from mice on 7 and 14 dpi (Figure 6). Regardless of treatment, all infected mice showed IgG responses against virus at 7 dpi compared to uninfected mice (Figure 6A). The levels of antibody production in serum collected from Tamiflu-, RG, and BG treated mice were increased two-fold at 14 dpi over that at 7 dpi (Figure 6B). These data indicate that treatment with BG does not interrupt virus-induced specific antibody production at the first virus inoculation.

### 3.6. BG Inhibits the Hemagglutination Activity of Influenza A Virus and Virus Replication in Vitro

**Figure 7:**
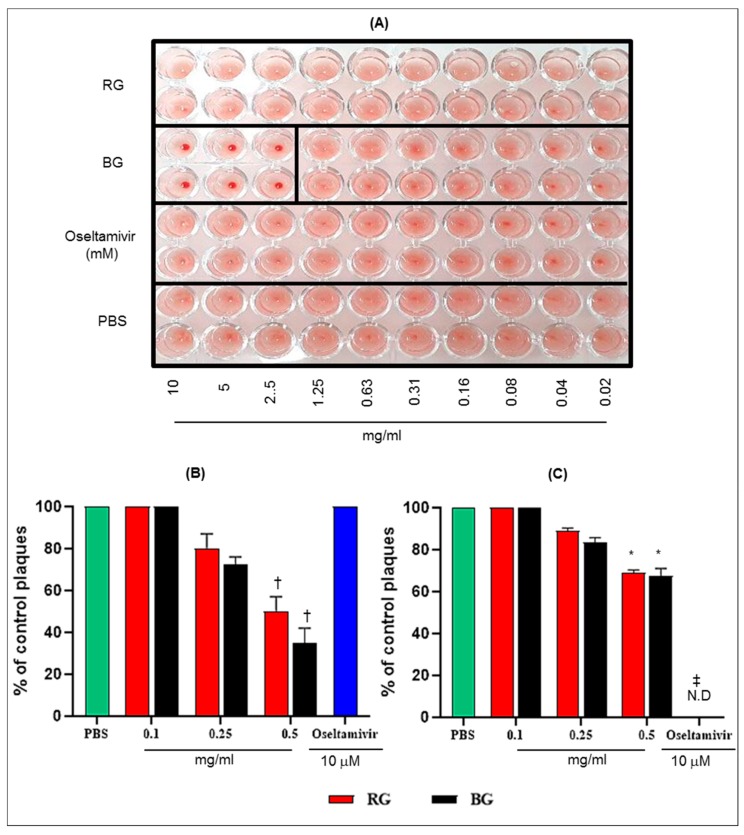
BG exhibits antiviral activities against A/California/04/2009 in vitro. (**A**) Hemagglutination inhibition (HI) assay conducted with PBS, RG, BG, and Oseltamivir with A/California/04/2009 in 96-well plates. A total of 0.02 to 10 mg/mL of each extract incubated with 4 to 8 HA unit of A/California/04/2009 virus for 60 min at RT and HI assay was conducted with 0.7% of turkey red blood cells (RBCs). Viral neutralization (plaque formation) assessments were performed in Madin-Darby Canine Kidney (MDCK) cells with (**B**) the pretreatment and (**C**) the posttreatment of BG against viral replication. The antiviral effect of BG was compared with RG and Oseltamivir treatment. Results are presented as the percentage of plaque reduction in each treatment group relative to the plaque formation in the PBS treatment group (negative control). Values are the mean ± SD *, *p* < 0.05 vs. PBS; †, *p* < 0.01 vs. PBS; ‡, *p* < 0.001 vs. PBS. N.D: Not detected.

To evaluate the direct effects of RG or BG treatment in mice on influenza A virus replication, we conducted HI assays. The HI assay results showed that the RG and Oseltamivir treatments could not inhibit the hemagglutination activity of A/California/04/2009 virus with red blood cells (RBC) (Figure 7A). However, at 2.5 to 10 mg/mL concentration, BG treatment could inhibit hemagglutination activity of A/California/04/2009 with RBC.

Further, we tested whether BG has antiviral activity in vitro by plaque reduction assay. It is noteworthy that pretreatment of MDCK cells with 0.5 mg/mL RG and BG reduce plaque formation by 50 and 65%, respectively, compared with the PBS-treated group (*p* < 0.01) (Figure 7B). Although plaque reduction was also observed, the posttreatment with RG and BG was less effective at reducing viral plaques (31% and 32.5%, respectively, at 0.5mg concentration) than their pretreatment (Figure 7C). Further, BG treatment resulted in greater plaque reduction activity than RG treatment under both conditions.

These results suggest that the antiviral effect of BG might be mediated through binding of the influenza virus particle and host innate immune responses following its pretreatment.

## 4. Discussion

The main purpose of this study was to examine the protective antiviral effects of BG (CJ EnerG) on respiratory pathogen-mediated immune dysfunction and mortality using mice infected with influenza A virus. To our knowledge, this is the first study to demonstrate that BG exhibits antiviral effects though the modulation of the immune system leading to host protection against lethal infection with influenza A virus.

Both innate immune responses, mediated by macrophages, dendritic cells and natural killer cells, and adaptive immune responses, mediated by T and B cells, occur following influenza A virus infection of the host [34]. Key molecules involved in this process are (1) IL-1β and TNF-α, pro-inflammatory cytokines that induce adhesion molecules for innate immune cells migrating to sites of infection, (2) IL-2, a T cell growth factor that stimulates T cell proliferation, (3) IFN-γ, produced by Th1 effector CD4 + T cells that regulates CD8 + T cell differentiation to clear the viral infection, and (4) IL-10, a negative regulator of inflammation that reduces host damage caused by pro-inflammatory cytokines during the recuperation phase of infection (reviewed in [35]). We demonstrated that BG induces the production of IL-2 and IFN-γ to amplify immune function, restrict viral replication, and euthanize virus-infected host cells upon viral infection. Moreover, during the recovery phase of infection, BG stimulates the production of IL-10 to decrease excessive immune activation and minimize potential host tissue damage. Interestingly, an immunomodulatory role of Rg3, a major ginsenoside of BG, has been identified. Administration of Rg3 was found to recover cyclophosphamide-induced immunosuppression by enhancing the production of IL-2 and IFN-γ and improving T cell production [36]. On the other hand, Rg3 was found to decrease the levels of pro-inflammatory mediators (e.g., TNF-α and IL-1β) and increase the production of anti-inflammatory cytokines (e.g., IL-10), resulting in attenuation of damage in lipopolysaccharide-induced acute lung injury in mice [37]. Thus, our data suggest that BG exerts potent immunomodulating properties.

Tamiflu inhibits neuraminidase on the surface of the virus, which prevents virus release from infected cells, thereby reducing viral replication and infectivity [10,38]. The most noticeable difference between the effects of BG and Tamiflu on mice infected with influenza A virus is the marked increase of GM-CSF in BALF induced by BG during the early stage of infection. The expression of GM-CSF in alveolar type II epithelial cells maintains immune homeostasis in the lung [39,40]. Further, the direct action of GM-CSF as an anti-influenza A virus agent has been confirmed in both genetically modified GM-CSF transgenic mice [32,41,42] and by intranasal administration of GM-CSF to mice [32,43]. Similar to our findings, GM-CSF was shown to modulate the immune system to reduce pneumonia by causing a dramatic decrease in immune cell infiltration into the lung during the late stage of infection [32,42]. Thus, unlike Tamiflu, the antiviral role of BG is mediated at least partially through the induction of GM-CSF, leading to decreased viral burden in the host.

In a mouse model of influenza virus infection, we demonstrated that treatment with BG induces a higher survival rate than treatment with RG, which was previously established as an antiviral agent [18,19,20,21,22,23,29]. Although the mechanism(s) underlying the antiviral effect conferred by BG is not clear yet, the HI assay demonstrates that only BG, and not RG, treatment can inhibit hemagglutination activity. Further, the plaque reduction assay revealed that pretreatment with BG attenuates A/California/04/09 virus replication in a dose-dependent manner with up to 65% plaque reduction in MDCK cells. These results suggest that the antiviral activity of BG might be associated with inhibition of hemagglutination activity and early induction of host innate immune responses. However, to elucidate the detailed mechanisms underlying the antiviral effects conferred by BG further studies are needed.

Ginseng contains various pharmacological components, including a series of tetracyclic triterpenoid saponins (ginsenosides), acidic polysaccharides, polyacetylenes, and polyphenolic compounds [44]. Previously, it was shown that treatment with acid polysaccharides extracted from RG results in increased mouse survival rates against influenza subtype H1N1 A/PR/8/34 in a dose-dependent manner (60% survival at 12.5 mg/kg bw dosage vs. 100% survival at 25 mg/kg bw dosage) [23]. Enhanced host protection against viral infection was also observed in mice following administration of saponins (67% survival) compared to administration of PBS (17% survival) [22]. Rb1, the most abundant ginsenoside in RG, has been shown to minimize viral activity in a dose-dependent manner, possibly through the mechanism that interfered with the attachment of viral hemagglutinin to sialic acid receptors on the surface of host cells [18]. Overall, acid polysaccharides and saponins independently play roles in protecting the host from viral infection. Thus, the increased effects of BG compared to those of RG on virus-associated respiratory pathogenesis may be due to the increased levels of more accessible acid polysaccharides in BG. However, whether the substantially different profiles of ginsenosides in BG and RG are responsible for the superior antiviral effects of BG remains to be determined.

## 5. Conclusions

We demonstrated that treatment with a novel BG (CJ EnerG) attenuates viral replication and lung histopathology by modulating immune responses, independent of IgG production, during infection. These effects lead to the protection of mice from lethal challenge with influenza A virus. Thus, BG may be a novel, orally active herbal adjuvant for the prophylactic treatment of influenza virus infections.

## Figures and Tables

**Table 1:** The amounts of ginsenosides in BG and RG.

	Acid Polysaccharides	Ginsenosides									
		Rb1	Rb2	Rc	Rd	Re	Rg1	Rg3(S)	Rk1	Rg5	Rh1
	m/g Extract										
RG ^1^	0.37	4.91	2.21	3.23	1.75	3.74	1.29	0.42	0.12	0.46	0.43
BG ^2^	2.63	0.83	ND ^3^	ND	ND	1.34	ND	4.12	4.75	4.54	0.94

^1^ RG: red ginseng; ^2^ BG: black ginseng; ^3^ ND: not detectable.
